# The Association between the Firmicutes/Bacteroidetes Ratio and Body Mass among European Population with the Highest Proportion of Adults with Obesity: An Observational Follow-Up Study from Croatia

**DOI:** 10.3390/biomedicines12102263

**Published:** 2024-10-04

**Authors:** Andrija Karačić, Ira Renko, Željko Krznarić, Sanja Klobučar, Ana-Marija Liberati Pršo

**Affiliations:** 1 The Gut Microbiome Center (CCM), 10110 Zagreb, Croatia or andrija.karacic@gmail.com (A.K.); ira@ccm.hr (I.R.); 2 Faculty of Food Technology and Biotechnology, University of Zagreb, 10000 Zagreb, Croatia; 3 University Hospital “Sveti Duh”, 10000 Zagreb, Croatia; 4 Department of Internal Medicine, School of Medicine, University of Zagreb, 10000 Zagreb, Croatia; zeljko.krznaric@kbc-zagreb.hr; 5 Department of Internal Medicine, Faculty of Medicine, University of Rijeka, 51000 Rijeka, Croatia; sanja.klobucar@uniri.hr; 6 Department of Endocrinology, Diabetes and Metabolic Diseases, University Hospital Rijeka, 51000 Rijeka, Croatia

**Keywords:** actinobacteria, Bacteroidetes, Firmicutes, F/B ratio, gut microbiome, gut microbiota, adiposity, obesity

## Abstract

**Background/Objectives**: The phyla Firmicutes and Bacteroidetes are the main constituents of the gut microbiota. An imbalance in the gut microbiota is a sign of dysbiosis, and the Firmicutes-to-Bacteroidetes ratio has been proposed to be a marker of it, especially in the context of obesity. Since Croatia is the country with one of the highest obesity rates in Europe, a pilot observational study was conducted. The aim of the study was to investigate the validity of this potential biomarker in a methodological study using sample processing, DNA sequence analysis and characterization of recruited participants, including various health factors. **Methods**: A study involving Croatian population was conducted. Participants age, body weight, gender, health history and lifestyle factors were recorded. Gut microbiota composition was analyzed using 16S rRNA sequencing. The F/B ratio was calculated and evaluated in the context of health factors. Statistical analysis was performed to detect the possible association of F/B ratio and excess body weight (kg) and possible impact of certain lifestyle factors. **Results**: No association between the F/B ratio and excess body weight (kg) was found. Excess body weight was significantly associated with higher age, male gender, and history of appendectomy. No significant health predictors of the F/B ratio were found, but weight gain was positively associated with a higher average F/B ratio. **Conclusions**: Although this study could not confirm the predictive value of the F/B ratio or any other phyla-related biomarker for excess body weight in the study population, it demonstrated interesting insights into the obesity-associated gut microbiota.

## 1. Introduction

The vast majority of bacteria constituting the gut microbiota belong to the two dominant phyla: the gram-positive Firmicutes and the gram-negative Bacteroidetes [1]. Only 10% of the gut microbiota is composed of members of other phyla, such as Actinobacteria, Proteobacteria, and Verrucomicrobia [2].

There is substantial interindividual variability regarding the relative abundances of the aforementioned phyla. This is due to internal and external factors—genetics [3], diet [4], physical activity [5], exposure to xenobiotics [6], climatic conditions [7], lifestyle, age, and geographic insights—that influence the gut microbiota composition by means of selective pressure. The high interindividual variability of the gut microbiota composition is an enormous challenge for research in this field [8]. The quest for the discovery of specific microbial signatures, especially in the context of disease states, is imminent [9].

In general, certain traits of gut microbiota composition have been established as signatures of troubled gut microbiota: reduced diversity and richness as well as an imbalance in the gut microbiota [10]. A distinct signature of microbial imbalance in the context of excess body weight, as proposed by a large number of scientific publications, is the ratio between the two dominant phyla of the gut microbiome: Firmicutes and Bacteroidetes [11]. The ratio between these two major phyla is abbreviated as the F/B ratio. Early work on the gut microbiota from research on animals and humans has shown that some overweight and obese subjects have increased abundances of Firmicutes at the expense of Bacteroidetes and thus a higher F/B ratio [12,13,14] compared with normal-weight subjects. Hence, the F/B ratio was brought forward as a potential biomarker.

Some studies have tried to attribute this increase to lifestyle and environmental factors that impact the composition of the gut microbiota [15,16], such as weight loss or gain, calorie-restricted diets, high-fat and high-fiber diets, and the Western diet (high intakes of pre-packaged foods, processed food, red meat, and high-fructose products) [12,13,14]. Nevertheless, increased values of the F/B ratio (values above 3.0) have been often linked to excess body weight. An increased F/B ratio has been associated with dysbiosis linked to obesity and concomitant metabolic and inflammatory disorders, such as diabetes, metabolic-associated fatty liver disease, gout, and dyslipidemia [17]. During the past decade, the F/B ratio has rapidly become a frequently cited microbial hallmark of excess body weight and especially obesity [17]. The phylum Firmicutes has been regarded as the more metabolically effective phylum [18], associated with a higher intake of calories, protein, fat, and sugar, and the Bacteroidetes phylum has been regarded as the metabolically ineffective phylum, associated with a higher intake of fiber [13,15,16,18,19,20,21].

However, over the past years, an increasing number of studies have shown contradictory results: either no association or even an inverse correlation between the F/B ratio and BMI. These findings have challenged the popular opinion of the F/B ratio as a biomarker of excess body weight [7].

A review by Magne et al. from 2020 concluded that the F/B ratio has no validity as a biomarker of obesity and excess body weight due to the plethora of lifestyle-associated factors influencing it (diet and physical activity) and the fact that technological aspects (sample processing, data and bioinformatic analysis, DNA extraction, choice of primers, and sequencing method) can heavily influence the identification of microbial abundances, overshadowing biological differences between samples [12]. Meta-analyses on data from obese subjects have shown that gut microbiota composition is clustered by study rather than BMI, indicating a substantial per-study effect [22]. This indicates that studies in general differ substantially in their methodologies when examining the variability of the obesity-associated gut microbiome. Additionally, the authors ascribed the heterogeneity in scientific findings regarding the F/B ratio to insufficient sample sizes in most studies on this topic. It has been estimated that approximately 160,000 individuals per group are required to detect a 1% difference in the F/B ratio, a far greater number than is seen in most studies [12,22,23,24].

Although the F/B ratio is well-noted biomarker, it is not the only proposed phyla-based biomarker of adiposity and obesity. Certain animal studies have pointed out that the difference in the microbiome composition on phyla level between obese and non-obese animals is only seen for the phylum Actinobacteria, whose members are represented in higher proportions among obese animals [25], with some evidence indicating this even in humans with obesity [26].

Another potential candidate for a biomarker of excess body weight is the phylum Proteobacteria. The Gram-negative bacteria in the phylum Proteobacteria are the most prominent members of the gut microbiota regarding endotoxic activity mediated by lipopolysaccharide production (LPS). This proinflammatory molecule is responsible for metabolic endotoxemia [12,25], which, in combination with low-grade inflammation, favors the development of obesity and related metabolic disorders, such as diabetes [27]. Increased proportions of Proteobacteria have been observed in humans and animals with obesity [12,28,29,30].

Obesity and related states associated with excess body weight are defined by an excessive accumulation of adipose tissue in the body with several consequent adverse effects on general health. Although this condition may be diagnosed in various ways, the most often used method in clinical practice is very simple and practical: the calculation of body mass index (BMI—body mass index or Quetelet’s index) [31]. Although its dietary regime is a combination of mid-European and Mediterranean, Croatia is the country most affected by the “obesity pandemic” in Europe [17]. A vast majority of the population has excess body weight (65%). Of these, 23% are living with obesity [17,32]. The Croatian government and healthcare system have been attempting to battle this issue with several national strategies [17] but with limited success. A novel perspective on the treatment of obesity based on Croatian data is urgently required to halt this perilous threat to public health.

Since the association between gut microbiota composition and BMI in Croatia has not been investigated to date, we decided to conduct an observational pilot study. The study was based on a similar study from Ukraine published in 2017 by Koliada et al., and the study design was based on this work to potentially reproduce the respective findings [25]. The aim was to confirm or refute the findings of the review by Magne et al. from 2020, which found no clear association between the F/B ratio and obesity [12]. The general purpose of this study was to assess the relevance of the F/B ratio as a marker of adiposity or excess body weight for utilization in the Croatian population. By first assessing the associations between the BMI and the relative abundances of major bacterial phyla, which has not been performed yet in the adult Croatian population, we attempted to evaluate the predictive value of the F/B ratio for adiposity and its potential role as a biomarker.

## 2. Materials and Methods

This study was conceived as an observational pilot study. The study was submitted to the ethical committee of the University Clinical Hospital Rijeka. The number of approval was 003-05/20-1/154.

### 2.1. Study Population

Participants were recruited from patients who admitted themselves to the Gut Microbiome Center, which is Croatia’s only institution specializing in gut microbiome analysis and research; it is in the capital but operates for patients in the whole country. All patients presenting for gut microbiome analysis irrespective of health complaints were potential candidates for this study.

The exclusion criteria were a history of oncological disease, inflammatory bowel disease, psychiatric disorders (substance use disorder, anxiety, depression, bipolar disorder, post-traumatic stress disorder, schizophrenia, disruptive behavior and dissocial disorders, eating disorders, and neurodevelopmental disorders), and administration of antibiotics or probiotics within the 3 months prior to study recruitment.

The sample size was established by power analysis using data from the Ukrainian study that this study design was based on. A minimum sample size of 125 individuals was determined to achieve statistical significance (*p* < 0.05) with a power of 0.8 for detecting differences between people with excess body weight and normal weight.

**Figure 1:**
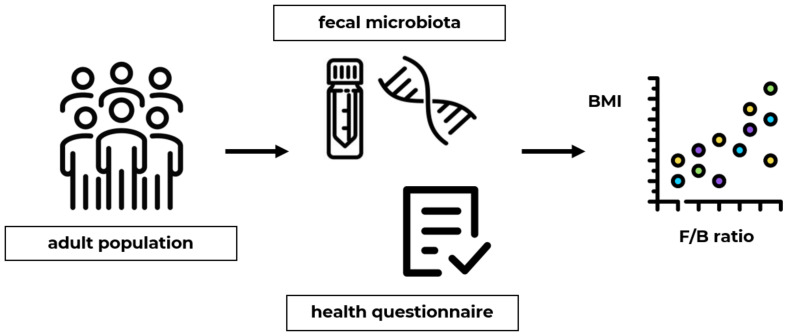
Study concept.

**Figure 2:**
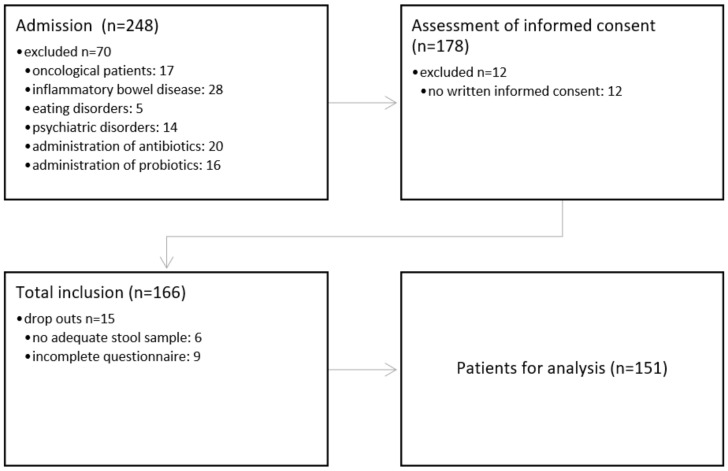
Study flow.

After an eight-month study enrollment phase (May 2023 to January 2024), a total of 151 adult volunteers were included in the study (Figure 1 and Figure 2).

These participants were grouped into three groups based on their BMI: those with a BMI < 18.5 kg/m^2^ (underweight), those with a BMI between 18.5 and 24.9 kg/m^2^ (normal weight), and those with a BMI ≥ 25.0 kg/m^2^ (overweight).

### 2.2. Sample Collection and 16s rRNA NGS of the Gut Microbiota

Fresh stool samples were provided by each participant in DNA buffer-containing vials. Participants took stool samples using a cotton swab of toilet paper at their home, following the instructions, and the swabs were conserved in up to 1000 µL of DNA-stabilizing buffer. The samples were transported by logistical services the next workday over the course of a couple of days to the laboratory (Biomes NGS, Wildau, Germany). Upon arrival, the stool samples were stored at −20 °C until sequencing. For the lysis process, the samples were defrosted and centrifuged at 4.000× *g* for 15 min. Afterward, 650 µL of warmed lysis buffer was added to each sample and then vortexed for 20 min. Afterward, the nucleic acids were extracted on a liquid handling system (Hamilton StarLine and Tecan EVO, London, UK) using a vacuum chamber as well as a high-pressure chamber. The extracted gDNA was stored at −20 °C until use. The library preparation followed the manual “16S Metagenomic Sequencing Library Preparation- Preparing 16S Ribosomal RNA Gene Amplicons for the Illumina MiSeq System”. For the normalization of all samples, a fluorescent dye and the Biotek Synergy HTX plate reader were used to measure DNA concentrations and to calculate the necessary dilution volume per sample. All the steps described were nearly fully automated by using a liquid handling system (Hamilton StarLine, London, UK), allowing for parallel sample processing. Library Denaturing and MiSeq Sample Loading was carried out manually following the Illumina protocol for the MiSeq Reagent Kit v3 (600-cycle). Demultiplexing was performed directly on the platform using MiSeq Reporter Analysis software right after sequencing, and the resulting FastQ files were generated for subsequent data analysis.

For the processing and analysis of sequence data, the Paired-End Reads from MiSeq (2 × 300 cycles) were merged to reconstruct overlapping sequences with a 430–460 base length. Chimera and borderline reads were filtered out with the usearch uchime2_ref tool 33. SILVA 138.1 was used as the database for usearch uchime2_ref. For taxonomic alignment, Amplicon sequence variants (ASVs) were determined using BLASTn (Nucleotide–Nucleotide BLAST 2.10.1+) (National Center for Biotechnology Information (NCBI), Bethesda, MA, USA) against SILVA 138.1 34. Alignment identity must meet the following thresholds: phylum: 75.0%, class: 78.5%, order: 82.4%, family: 86.5%, genus: 94.5%, and species: 97.0%. RefSeqs/Counts tables were created for all samples using the Python package Pandas 1.3.4. The taxonomic composition of microbial communities was inferred from ASV (amplicon sequence variants) counts at the phylum, genus, and species levels. Further bioinformatic analyses were performed using Picrust2 35. The study sequences of the alignment step were placed into a reference tree to determine/predict the copy numbers and the NSTI (nearest-sequenced taxon index). All study sequences with an NSTI higher than 2 were excluded.

### 2.3. Primers

The gene-specific sequences used in this protocol targeted the 16S V3 and V4 regions. Illumina adapter overhang nucleotide sequences were added to the gene-specific sequences. The full-length primer sequences, using standard IUPAC nucleotide nomenclature and following the protocol targeting this region, were as follows: 16S Amplicon PCR Forward Primer = 5′

TCGTCGGCAGCGTCAGATGTGTATAAGAGACAGCCTACGGGNGGCWGCAG

16S Amplicon PCR Reverse Primer = 5′

GTCTCGTGGGCTCGGAGATGTGTATAAGAGACAGGACTACHVGGGTATCTAATCC

The overhang adapter sequences that had to be added to the locus-specific primer for the region to be targeted were as follows:

Forward overhang: 5′ TCGTCGGCAGCGTCAGATGTGTATAAGAGACAG-[locus-specific sequence]

Reverse overhang: 5′ GTCTCGTGGGCTCGGAGATGTGTATAAGAGACAG-[locus-specific sequence].

### 2.4. Statistical Analysis

Statistical analysis was performed in an integrated development environment for the R programming language, R Studio. To test the normality of the distribution of all the quantitative variables, the Shapiro–Wilk test was used. Since the variables did not follow a normal distribution, non-parametric methods were selected for further analysis of the data, such as Spearman’s correlation and multivariate logistic regression. To identify the statistical differences between the BMI categories, the median abundances of each phylum were compared by the Kruskal–Wallis test.

The adjustment for these factors was performed by a multivariate logistic regression model utilizing the integrated development environment for R programming language, R Studio, along with the F/B ratio.

## 3. Results

The demographic and lifestyle characteristics of studied participants are presented in Table 1. Most participants were female, and the age group of 20–39 years old was the most represented. Regarding their lifestyle factors, 77% of all participants were non-smokers, and 70% of them had not experienced any changes in their body mass in the past few years. Regarding dietary habits, it is interesting that 44% of all participants were trying to watch their fiber intake (Table 1). Regarding supplement intake, in general, the number of participants who were not taking supplements or some sort of drug was greater than those who were (Table 1).

The median F/B ratio was 3.11, with an IQR of 2.35–4.47. The average BMI was 23.08 kg/m^2^, with an IQR of 20.35–25.95 kg/m^2^ (Table 2 and Table 3). The median Firmicutes/Bacteroidetes/Proteobacteria/Actinobacteria/Verrucomicrobia abundances were 67.48/21/1.9/3/0.1, with IQRs of 61.85–73.00/16–26/0.83–3.82/1.85–5.75/0.006–0.655, and the range of the detected abundances (minimum, maximum) are shown in Table 2.

**Figure 3:**
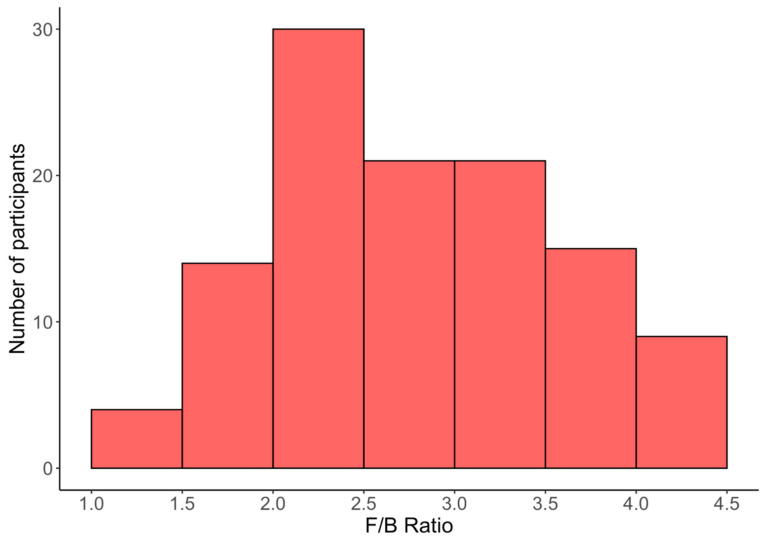
Distribution of F/B ratio values in the study population for most participants (F/B ratio < 4.5).

The frequency of F/B ratio values in the study population is shown in Figure 3. The vast majority—more than 70%—of the study population had an F/B ratio between 2.0 and 3.5, the most prominent being values between 2.0 and 2.5.

**Figure 4:**
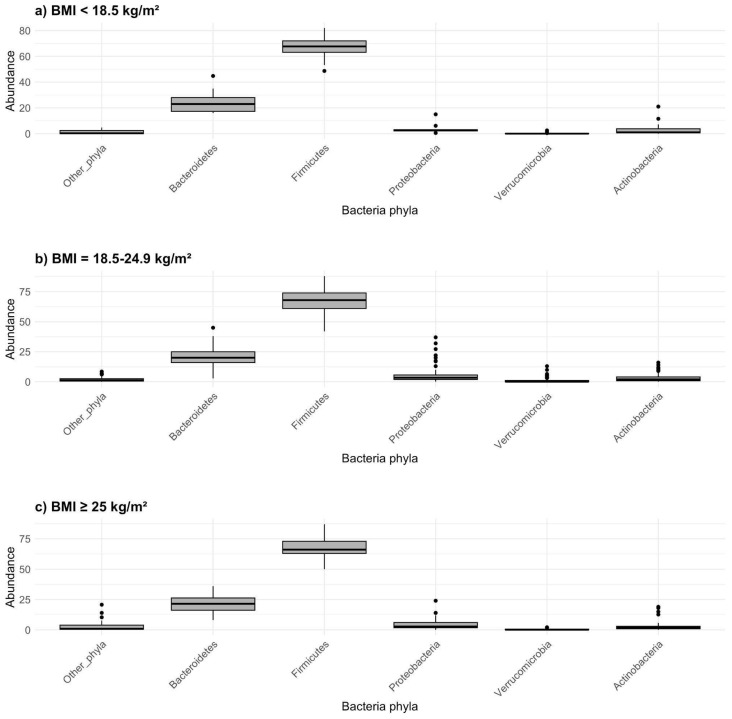
Gut microbiome composition on the phyla level in the context of BMI: (**a**) BMI < 18.5 kg/m^2^, (**b**) BMI = 18.5–24.9 kg/m^2^, (**c**) BMI ≥ 25 kg/m^2^. Abundance is defined by median and quartile values for each bacteria phylum.

**Figure 5:**
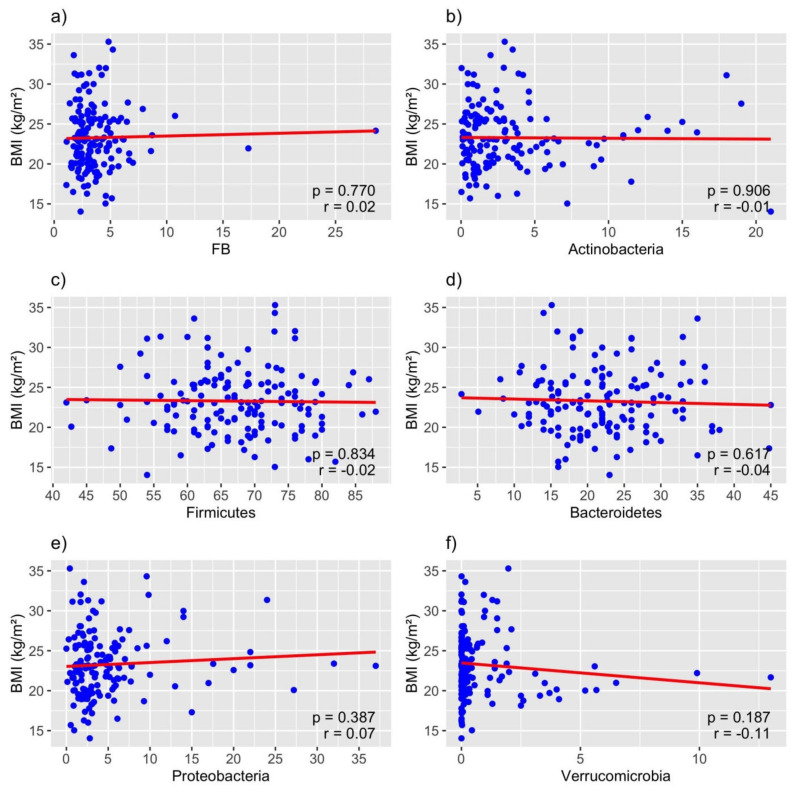
Regression plots of BMI against relative proportions of phyla and (**a**) the Firmicutes/Bacteroidetes ratio (FB), (**b**) Actinobacteria, (**c**) Firmicutes, (**d**) Bacteroidetes, (**e**) Proteobacteria, and (**f**) Verrucomicrobia; r: Spearman’s correlation coefficient.

The relative abundances of the major microbial phyla varied between the different BMI categories (Table 3, Figure 4 and Figure 5). No consistent trends were seen between BMI groups regarding the different phyla. The abundance of Firmicutes was almost identical in all three groups, but the median abundance of the phylum Bacteroidetes was lower in the overweight group than in the underweight group. The greatest differences regarding median phylum abundance were seen in the phylum Verrucomicrobia, with the median value being a hundred times smaller in the underweight group than in the other two. However, these differences between groups were not statistically significant, probably due to the minuscule difference between the normal weight and overweight groups as well as the small sample size. To identify statistical differences between the BMI categories, median values were compared using the Kruskal–Wallis test, which yielded no significant results (Table 3). To assess the potential association of alternative ratios and body weight, the Firmicutes-to-Proteobacteria ratio and Proteobacteria-to-Verrucomicrobia ratio were calculated and then compared between the three groups. Neither alternative ratio was found to be significantly different between groups.

Additionally, in a univariate unadjusted logistic regression model, the F/B ratio was also not significantly associated with BMI (*p* = 0.705).

To assess the role of other covariates composed by the present study on the BMI besides the F/B ratio, an inferential statistical analysis was performed. Differences in the only quantitative variable (age) and the qualitative variables (gender; smoking; change in body mass; appendectomy; alcohol consumption; dietary regime; fiber; probiotic, antibiotic, and supplement intake before three months; chronic medication; and physical activity) were assessed between the BMI groups together with the F/B ratio using a multivariate logistic regression model. Excess body weight was correlated with age (*p* < 0.001), gender (*p* < 0.001), a history of appendectomy (*p* = 0.049), medicament consumption (*p* = 0.02), and physical activity (*p* = 0.02). No statistically significant differences were seen for the F/B ratio (*p* = 0.676), smoking (*p* = 0.456), dietary regime (*p* = 0.368), change in body mass (*p* = 0.862), probiotic intake (*p* = 0.420), antibiotic intake (*p* = 0.682), supplement intake (*p* = 0.629), alcohol consumption (*p* = 0.577), or fiber intake (*p* = 0.215).

To better assess the influence of the covariates assessed in the present study on the F/B ratio inferential statistical analysis was performed (Kruskal–Wallis, Mann–Whitney). The results are disclosed in Table 4. No statistically significant difference was observed regarding the F/B ratio at the level of any variable (Table 4). It was found that subjects who reported weight gain had a higher F/B ratio than those who reported weight loss, but the difference was not found to be significant.

## 4. Discussion

The main finding of our study is that no correlation between the F/B ratio and the BMI was found. In our study, the F/B ratio had no predictive value regarding BMI. Although these results are in accordance with a great number of other studies [22,33,34], the results differ from those of the original study by Koliada et al. [35], who found a significant association between the F/B ratio and BMI, a finding reproduced in several studies [28,29,36].

Furthermore, the results of the multivariate analysis showed that higher age, male gender, and history of appendectomy were statistically significant independent predictors of excess body weight when assessed together with the F/B ratio. The fact that a history of appendectomy is a significant predictor of excess body weight reinforces the role of the gut microbiota in adiposity and obesity. An appendectomy leads to profound changes in the gut microbiota [37,38], impacting insulin regulation.

Since the proportions of the other two potential phyla-based markers, Actinobacteria and Proteobacteria, were also not correlated with BMI, they were also not found to be adequate biomarkers of excess body weight. Two suggested potential alternatives to the F/B ratio (the Firmicutes-to-Proteobacteria and Proteobacteria-to-Verrucomicrobia ratios) were also not found to be correlated with greater BMI.

Also, no health factors included in this study were found to be significantly correlated with the F/B ratio. It must be noted that the F/B ratio was higher among participants who reported weight gain than those who reported weight loss, and this association could have even been potentially significant in a larger study sample (*p* = 0.09). Curiously, this finding is in line with a study linking higher Firmicutes proportions to weight gain in childhood [39].

For a more substantial discussion on the study results, they were compared with a review of nine observational studies from around the globe conducted by Magne et al. in 2020 as well as a large-scale study from Japan [40] with around 6000 participants of all ages. Although the median BMI was like that in most of these studies, our study population was younger, comparable with only three of the ten analyzed study populations. Our results regarding the F/B ratio and the abundances of the two dominant phyla differed greatly; the F/B ratio was higher than that in any other population, the median being closest to the ones from the populations from the USA and UK. This is primarily attributable to the high proportions of Firmicutes, which were similar only to those of the American and British populations and far from the values of the Japanese, Indian, and Pakistani populations. This could reinforce the hypothesis that an elevated F/B ratio is a consequence of urbanization and the Western diet, as seen in African populations [39] due to the phylum Firmicutes.

Species belonging to phylum Firmicutes indeed preferentially use refined foods as growth substrates [40]. However, unfortunately, it seems that our sample was not suitable for such a generalization, since, for example, the percentage of vegetarians and vegans in our sample was much greater than that in the general population (6.7% vs. 3.7%) [40,41]. The inadequacy of our sample for generalization in Croatia is far more drastic when assessing the proportion of individuals with overweight and obesity; 31% of the study participants were overweight, whereas 65% of Croatians were overweight in 2023 (Table 1). Our study sample is just much thinner than the Croatian general population. Croatians are increasingly consuming a Westernized diet and adhering less and less to their traditional Mediterranean diet [42,43], which has definitely contributed to making Croatia the European forerunner regarding obesity. Maybe the relatively high F/B ratio indicates that the sample was recruited from a population mostly eating a Westernized diet, but since they are younger than participants in other studies, our participants have just not yet developed excess body weight.

## 5. Conclusions

The data obtained in our study indicate that there is no significant positive correlation between either higher Firmicutes levels or F/B ratio values and BMI and hence excess body weight. The results suggest that in younger, more health-conscious populations, the F/B ratio is not associated with greater BMI and obesity, and therefore, it is not a robust biomarker of excess body weight. The present study is limited due to study design and study execution. The recruitment process was heavily biased by several selection biases (volunteer bias, non-response bias, and undercoverage bias) since health-aware people are more likely to respond and volunteer in this type of study than people who are overweight or obese. Due to technical constraints, the recommendation of the authors of the original study to perform sampling from the small intestine could not be realized.

Considering earlier projections [20,22,23,24], our study lacks the power to detect modest differences in the F/B ratio. However, this in a way confirms our study findings that the F/B ratio lacks robustness for the assessment of dysbiosis associated with excess body weight since it is not reliable in populations with a significant number of normal-weight people.

It is important to note that BMI itself is not a good measure of excess body weight and thus adiposity and obesity since it does not take muscle mass into account. BMI is not an indicator of cardiometabolic health but rather a simple diagnostic instrument for the evaluation of excess body weight. Body composition evaluations would be a better solution for future studies on this topic since it has been demonstrated that cardiometabolic health is a good discriminator of gut microbiota composition [12,44].

Another limitation of this study is that the participants self-reported their body mass, and the anthropometric parameters were not evaluated by the researchers themselves. Although great effort was put into the characterization of participants, due to technical constraints and the employment of a self-report instrument, self-report bias is present.

Our results can hardly be generalized to other populations, especially in different geographical locations, since gut microbiota signatures differ around the world depending on geographical location [12,45,46,47,48].

The strength of our study is that it is the first work on the association between the F/B ratio and gut microbiome composition in general and adiposity in Croatia. Our findings could confirm the hypothesis that the concept of the F/B ratio as a unique taxonomic signature of excess body weight is not valid, and that the F/B ratio has no validity as a potential biomarker. On top of that, it was modeled upon an existing, published observational study whose sample size was exceeded in the present study.

For future studies, we plan to include a more diverse population, especially regarding BMI and lifestyle choices, by offering incentives such as financial compensation. The issues of misrepresentation should be addressed in future research. A greater sample size is required for high-quality conclusions, as mentioned before.

## Figures and Tables

**Table 1:** Demographic and lifestyle characteristics.

Variable	BMI Category			
	<18.5 kg/m^2^ underweight N (% in total)	18.5–24.9 kg/m^2^ normal weight N (% of total)	≥25 kg/m^2^ overweight N (% of total)	Total N (% of total)
Age:				
<20	3 (2%)	1 (1%)	-	4(3%)
20–39	6 (4%)	58 (38%)	23 (15%)	87 (57%)
39–59	3 (2%)	29 (19%)	22 (15%)	54 (36%)
>59	1 (1%)	2 (2%)	3 (3%)	6 (4%)
Gender:				
Male	1 (1%)	25 (17%)	21 (14%)	47 (32%)
Female	12 (8%)	65 (43%)	27 (17%)	104 (68%)
Tobacco smoking:				
Never	10 (7%)	66 (44%)	39 (26%)	115 (77%)
Rarely (1–2 cigarettes per week)	1 (1%)	14 (9%)	4 (3%)	19 (13%)
Occasionally (3–4 cigarettes per week)	1 (1%)	7 (4%)	-	8 (5%)
Every day	1 (1%)	3 (2%)	5 (3%)	9 (6%)
Change in body mass:				
No change	8 (5%)	67 (44%)	30 (21%)	105 (70%)
Increase in body mass	4 (3%)	10 (7%)	5 (3%)	19 (13%)
Decrease in body mass	1 (1%)	10 (7%)	16 (10%)	27 (18%)
Appendectomy:				
No	12 (8%)	85 (56%)	42 (28%)	139 (92%)
Yes	1 (1%)	5 (3%)	6 (4%)	12 (8%)
Dietary regime:				
Omnivore	10 (7%)	70 (46%)	41 (27%)	121 (80%)
Vegetarian/vegan	2 (1%)	7 (5%)	1 (1%)	10 (7%)
Other	1 (1%)	15 (10%)	4 (2%)	20 (13%)
Dietary fiber intake as priority:				
Yes	1 (1%)	28 (18%)	12 (8%)	41 (27%)
No	5 (3%)	23 (15%)	15 (10%)	43 (28%)
I am trying to	7 (4%)	39 (26%)	21 (14%)	67 (44%)
Physical activity:				
Never	-	8 (5%)	4 (3%)	12 (8%)
Rarely (1–2 times per week)	2 (1%)	28 (18%)	22 (15%)	52 (34%)
Frequently (3–4 times per week)	10 (7%)	38 (25%)	13 (9%)	61 (41%)
Every day	1 (1%)	16 (10%)	9 (6%)	26 (17%)
Antibiotic intake before 3 months				
Yes	-	2 (1%)	1 (1%)	3 (2%)
No	13 (9%)	88 (58%)	47 (31%)	148 (98%)
Drugs				
Yes	3 (2%)	29 (19%)	22 (15%)	54 (36%)
No	10 (7%)	61 (40%)	26 (17%)	97 (64%)
Probiotic intake before 3 months				
Yes	5 (3%)	29 (19%)	13 (9%)	47 (31%)
No	8 (5%)	61 (41%)	35 (23%)	104 (69%)
Dietary supplement intake				
Yes	10 (7%)	63 (42%)	32 (21%)	105 (70%)
No	3 (2%)	27 (18%)	16 (10%)	46 (30%)
Alcohol consumption				
Not at all	7 (5%)	12 (8%)	5 (3%)	24 (16%)
Rarely	5 (3%)	41 (27%)	20 (13%)	66 (43%)
Occasionally	1 (1%)	35 (23%)	22 (14%)	58 (38%)
Often	-	2 (1%)	1 (1%)	3 (2%)
Total	13 (8%)	90 (60%)	48 (32%)	151

N: number of participants.

**Table 2:** Data on different bacterial phyla.

Phylum				
	Median	IQR	Minimum	Maximum
Firmicutes	67.48	61.85–73	42	88
Bacteroidetes	21	16–26	2.8	45
Actinobacteria	1.9	0.83–3.82	0.03	21
Proteobacteria	3	1.85–5.75	0	37
Verrucomicrobia	0.096	0.006–0.655	0	13

**Table 3:** The abundance of the phyla in the context of BMI representing the median, quartiles, and values obtained with the Kruskal–Wallis test.

Phylum	BMI Category				Kruskal–Wallis Test		
	Underweight (<18.5 kg/m^2^)	Normal Weight (18.5–24.9 kg/m^2^)	Overweight (≥25 kg/m^2^)	Total	χ2	df	*p*-Value
Firmicutes	68 (63–72)	68 (61–74)	66 (63–73)	67.5 (62–73)	0.031	2	0.985
Bacteroidetes	23 (17.26–28)	20.9 (16–25)	21.5 (16.16–26.31)	21 (16–26)	1.323	2	0.516
Actinobacteria	1.03 (0.61–3.8)	2.0 (0.85–4.05)	1.75 (0.893–2.95)	1.9 (0.83–3.82)	0.408	2	0.815
Proteobacteria	2.72 (2.1–3.06)	3.4 (1.9–5.7)	2.77 (1.77–6.10)	3 (1.85–5.75)	1.90	2	0.387
Verrucomicrobia	0.01 (0.004–0.097)	0.12 (0.015–1.1)	0.10 (0.004–0.658)	0.096 (0.006–0.655)	3.671	2	0.160
Other phyla	0.29 (0.00–2.43)	1.30 (0.603–2.597)	0.94 (0.406–3.898)	1.06 (0.495–2.885)	4.871	2	0.088
F/B ratio	2.92 (2.35–3.79)	3.14 (2.38–4.48)	3.12 (2.38–4.48)	3.11 (2.358–4.469)	0.765	2	0.682
F/P ratio	23.23 (21.92–33.33)	20.29 (11.46–35.33)	23.93 (11.13–39.23)	22.70 (11.27–36.78)	1.126	2	0.570
P/V ratio	33.96 (6.99–250.34)	16.84 (2.93–180)	19.64 (5–180.36)	20.8 (4.55–192.86)	0.289	2	0.865

F: Firmicutes; B: Bacteroidetes; P: Proteobacteria; V: Verrucomicrobia.

**Table 4:** Impact of covariates on F/B ratio.

Variable					Kruskal–Wallis Test		
					χ2	df	*p*-Value
Age							
years	<20	20–39	39–59	>59			
F/B ratio (median, IQR)	3.2 (2.2–4.3)	3.1 (2.3–4.5)	3.1 (2.4–4.3)	3 (2.6–3.5)	0.349	3	0.951
Gender							
	male	female					
F/B ratio (median, IQR)	3(2.3–4.5)	3.1 (2.4–4.5)			0.242	1	0.623
Tobacco smoking							
	No	Rarely	Occasionally	Often			
F/B ratio (median, IQR)	3.1 (2.4–4.3)	3.2 (2.7–4.8)	2.6 (2–3.1)	3.4 (2.6–4.5)	2.267	3	0.519
Change in body mass							
	No change	Increase in body mass	Decrease in body mass				
F/B ratio (median, IQR)	3.2 (2.4–4.7)	3.5 (2.3–4.5)	2.9 (2.2–3.1)		4.676	2	0.097
Appendectomy							
	Yes	No					
F/B ratio (median, IQR)	3.1 (2.4–4.5)	3.1 (2.4–3.9)			0.006	1	0.937
Dietary regime							
	Omnivore	Vegetarian/vegan	Other				
F/B ratio (median, IQR)	3.2 (2.4–4.6)	3.2 (2.4–4.8)	2.8 (2.2–3.2)		1.822	2	0.402
Dietary fiber intake as priority							
	Yes	No	I am trying				
F/B ratio (median, IQR)	3.1 (2.4–4.6)	3.2 (2.5–4.1)	3.1 (2.2–4.6)		0.352	2	0.839
Physical activity							
	Never	Rarely (1–2× per week)	Frequently (3–4× per week)	Every day			
F/B ratio (median, IQR)	3.2 (2.8–4.2)	3.2 (2.3–4.1)	3 (2.3–4.5)	3.3 (2.6–4.6)	1.816	3	0.612
Antibiotics (before 3 months)							
	Yes	No					
F/B ratio (median, IQR)	2 (1.9–2.7)	3.1 (2.4–4.5)			2.231	1	0.135
Chronic medication							
	Yes	No					
F/B ratio (median, IQR)	3.1 (2.4–4)	3.2 (2.3–4.6)			0.146	1	0.7022
Probiotics (before 3 months)							
	Yes	No					
F/B ratio (median, IQR)	3.1 (2.4–4)	3.1 (2.3–4.6)			0.005	1	0.946
Dietary supplementation							
	Yes	No					
F/B ratio (median, IQR)	3.1 (2.4–4.4)	3.2 (2.2–4.5)			0.128	1	0.720
Alcohol intake							
	Never	Occasionally	Regular				
F/B ratio (median, IQR)	3.3 (2.4–4.6)	3 (2.5–4.2)	3.2 (2.3–4.7)		0.269	2	0.874

## Data Availability

The data presented in this study are available upon request from the corresponding author due to ethical and medicolegal reasons. The metagenomic sequences will not be deposited in a public database. The results of sequencing will be available only upon request to the author.

## Abbreviations

BMI: Body mass index; F/B ratio: Firmicutes/Bacteroidetes ratio; PAL: Physical activity level; qRT-PCR: Quantitative real-time PCR
